# Reference Values of Noninvasive Myocardial Work Indices Measured by Echocardiography in Healthy Children

**DOI:** 10.3389/fped.2022.792526

**Published:** 2022-06-16

**Authors:** Cunying Cui, Qiang Zheng, Yanan Li, Danqing Huang, Yanbing Hu, Ying Wang, Rujie Liu, Lin Liu, Lianzhong Zhang

**Affiliations:** ^1^Department of Ultrasound, People's Hospital of Zhengzhou University, Zhengzhou, China; ^2^School of Computer and Control Engineering, Yantai University, Yantai, China; ^3^Department of Ultrasound, The First Affiliated Hospital of Zhengzhou University, Zhengzhou, China

**Keywords:** pediatric, reference value, echocardiography, myocardial work, normal value

## Abstract

**Backgroud:**

Noninvasive myocardial work, estimated by left ventricular (LV) pressure-strain loop (PSL), has been introduced for assessing LV myocardial performance. Based on both blood pressure and speckle-tracking derived strain data, noninvasive myocardial work is considered to be less load-dependent than global longitudinal strain (GLS). In some conditions, such as hypertension or aortic coarctation, the increased afterload will affect strain measurements, and myocardial work can serve as a more robust metric.

**Objective:**

We prospectively recruited healthy children to explore the relationship between myocardial work indices and body size parameters, and to determine the reference values of noninvasive myocardial work indices in healthy children.

**Methods:**

183 healthy children (aged 1–18 years, males: 52.5%) were enrolled in the study. Global work index (GWI), global constructive work (GCW), global wasted work (GWW), global work efficiency (GWE), were assessed by LVPSL and compared according to age and sex.

**Results:**

The mean for GWI was 1,448.7 ± 265.0 mm Hg%, 1,859.8 ± 290.7 mm Hg% for GCW, and the median (interquartile range) for GWW was 54.0 (33.0–82.0) mm Hg% and 97.0 (95.0–99.0) % for GWE. male had greater GWI and GCW) than female (1,572.5 ± 250.2 mm Hg% vs. 1,312.2 ± 208.7 mm Hg% and 1,944.3 ± 299.2 mm Hg% vs. 1,766.6 ± 251.5 mm Hg%, respectively, all *P* < 0.001). GWI and GCW were significantly correlated with baseline parameters, including age, height, weight, BSA, body mass index, heart rate, and blood pressure. After indexed to BSA, GWI (BSA), GCW (BSA) remained significantly negatively correlated with age (*P* < 0.001).

**Conclusions:**

we proposed the normal reference values and regression equations for GWI and GCW based on age and BSA in healthy children. This might provide a basis of reference for the evaluation of cardiac function in children with cardiopulmonary disease.

## Introduction

Noninvasive myocardial work, estimated by left ventricular (LV) pressure-strain loop (PSL), has been introduced as an echocardiographic parameter for assessing LV myocardial performance (1–4). Based on both blood pressure and speckle-tracking derived strain data, noninvasive myocardial work is considered to be less load–dependent than global longitudinal strain (GLS)(5). In some conditions, such as hypertension or aortic coarctation, the increased afterload will affect strain measurements, and myocardial work can serve as a more robust metric. Moreover, it has been used to evaluate LV systolic function in various cardiovascular diseases (6–9). However, there are few studies in children, and the parameter lacks reliable reference values in pediatric populations.

Previous studies have provided adult and pediatric reference values of myocardial work indices (10–14). But the findings on the correlations of age and sex with myocardial work indices were inconsistent. In the present study, we prospectively recruited healthy children to further explore the relationship between myocardial work indices and body size parameters, including age, weight, height and body surface area (BSA), and to determine the reference values of noninvasive myocardial work indices in healthy children.

## Methods

### Study Population

**Figure 1:**
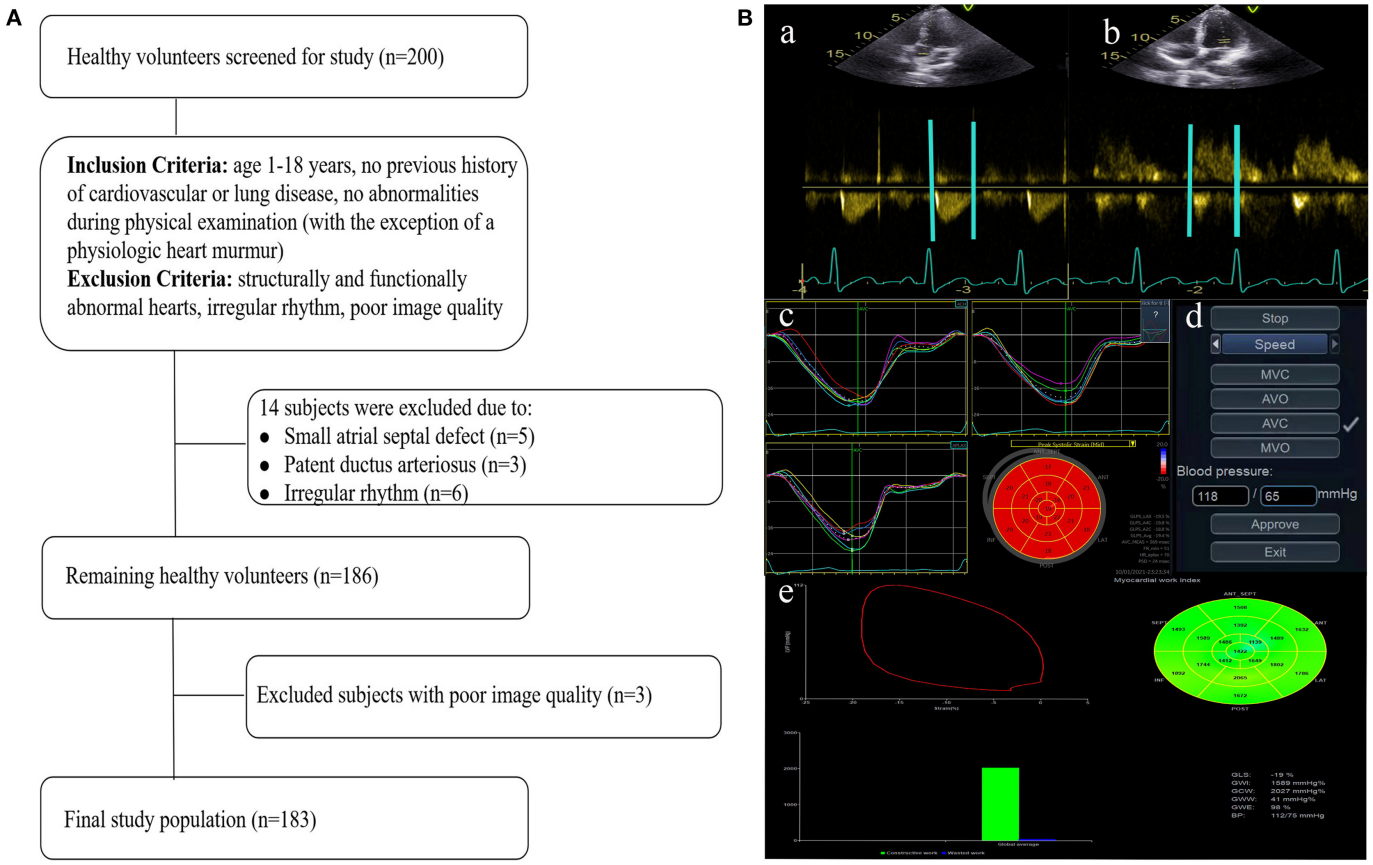
Flow chart detailing the identification of the study cohort **(A)** and image analysis for myocardial work indices **(B)**. Eventing time was determined based on the Doppler flow spectrum of the aortic valve (a) and mitral valve (b). Tracking was automatically performed to determine segmental and global longitudinal strain (c). After entering the cuff blood pressure value (d), the software automatically generated the PSL curve and myocardial work indices (e). The lower right corner of figure e displays the myocardial work indices obtained from the PSL curve. PSL, pressure-strain loop.

This prospective study was performed in the ultrasound department of Fuwai Central China Cardiovascular Hospital. A total of 200 healthy children (aged 1–18 years), who were recruited from local schools or from an outpatient clinic for health examination, were enrolled in the study. The inclusion and exclusion criteria are listed in Figure 1A. The inclusion criteria were: age 1–18 years; no previous history of cardiovascular or lung disease, and no abnormalities during physical examination (with the exception of a physiologic heart murmur). Subjects with structurally and functionally abnormal hearts, including minor defects such as small atrial septal defect, patent ductus arteriosus, or irregular rhythm, or subjects with images of poor quality were excluded. Of the 200 healthy volunteers enrolled, 17 volunteers were excluded due to small atrial septal defect (*n* = 5), patent ductus arteriosus (*n* = 3), irregular rhythm (*n* = 6), and poor image quality (*n* = 3). The final analysis included 183 volunteers. This study was approved by the ethics committee of Fuwai Central China Cardiovascular Hospital and written informed consent was obtained from each subject or their parents/guardians.

Age, sex, height, weight, heart rate (HR) and blood pressure of each subject were recorded during the study. BSA was calculated according to the formula proposed by Haycock et al. (15): BSA (m^2^) = weight (kg) ^0.5378^ × height (cm) ^0.3964^ × 0.024265. Body mass index (BMI, kg/m^2^) was calculated as weight (kg)/height (m)^2^. BMI was corrected for age and sex according to World Heart Organization standards (16, 17). Those with corrected BMI outside the 2nd or 98th percentile were excluded.

### Echocardiographic Image Acquisition

All echocardiographic examinations were performed using a Vivid E95 ultrasound system (GE vingmed ultrasound, Horten, Norway) equipped with an M5Sc-D 1.4–4.6 MHz transducer. The subjects were examined in left lateral position. No sedative was used. After synchronously connecting the electrocardiogram, all images were collected at resting state. Using a standardized echocardiographic functional protocol, all subjects underwent a comprehensive echocardiographic examination. Grayscale images of standard apical four-, three- and two- chamber views were collected at frame rates between 40 and 90 frames per second. A minimum of three cardiac cycles were collected for each view. Doppler flow spectra of aortic valve and mitral valve were collected in apical five- and four- chamber views, respectively. Blood pressures were measured by placing a cuff over the brachial artery immediately following acquisition of the apical images to capture the hemodynamic state during image acquisition. Blood pressures were presented as the average of 2 to 3 readings. HR during blood pressure measurement and image acquisition was similar. All images were stored in a digital raw data format (native DICOM format) for offline analysis of myocardial work indices.

### Myocardial Work Analysis

Analysis was performed according to the vendor's instructions and described by previous studies (18, 19). Echopac version 203 (GE vingmed ultrasound, Horten, Norway) was used for image analysis. Eventing time was determined based on the Doppler flow spectrum of the aortic valve and mitral valve. Tracking was automatically performed. If the tracking was not satisfying, tracking points were adjusted manually to determine segmental and global longitudinal strain. After entering the cuff blood pressure value, the software automatically generated the PSL curve and the following myocardial work indices (Figure 1B):

- Global work index (GWI), the mean of segmental myocardial work index, which represents the total performance from mitral valve closure to mitral valve opening.
- Global constructive work (GCW), work performed during shortening in systole, adding negative work during lengthening in isovolumetric relaxation.
- Global wasted work (GWW), negative work performed during lengthening in systole, adding work performed during shortening in isovolumetric relaxation.
- Global work efficiency (GWE), the percentage of constructive work, which is the sum of constructive work and wasted work.

To evaluate intra-observer variability, images of 20 subjects were randomly selected and analyzed twice by one person with an interval of >2 weeks. For inter-observer consistency, the same images were analyzed by a second person who was blinded from the previous results.

### Statistical Analysis

All statistical analyses were performed using SPSS version 20 (SPSS Inc., Chicago, IL, USA). Normality of the distribution of continuous variables was assessed by the Kolmogorov-Smirnov test. Normally distributed, continuous variables were expressed as mean ± standard deviation. For normally distributed variables, comparisons between males and females were analyzed by the unpaired *t*-test, and comparison according to age groups (1–3, 4–6, 7–9, 10–12, 13–15, and 16–18 years) was done by the one-way analysis of variance test. Non-normally distributed, continuous variables were expressed as median (interquartile range). For non-normally distributed variables, the Mann-Whitney test was used for comparisons between males and females, and comparison according to age groups was done by the Kruskal-Wallis test pairwise comparisons. Categorical variables were presented as number (%). Associations between myocardial work indices and baseline characteristics were analyzed by Pearson's or Spearman's correlation coefficients. Scatter diagrams of myocardial work parameters with age were plotted as trend curves generated from weighted regression. Bland-Altman plots were used to test the intra- and inter-observer variability. Interclass coefficients (ICCs) and 95% confidence intervals (CIs) were calculated. A *P* value < 0.05 was considered statistically significant.

### Sample Size

Sample size was estimated using our preliminary results on mean differences between male and female myocardial work indices for the whole population, we compared the mean myocardial work indices in six age groups to calculate the sample size of each subgroup. We assumed a 20% dropout rate. To detect a significant effect with 90% statistical power (a = 0.05), recruitment of 130 participants (65 males and 65 females) and no <14 participants in each age group was needed.

## Results

### Baseline Characteristics

As depicted in Figure 1A, 183 healthy participants were included in the final analysis, 96 males and 87 females (47.5%). The subjects' characteristics are summarized in Table 1. The median age was 10.0 years (25th−75th: 6.0–13.0 years). Males presented with greater height, weight, BSA, and higher blood pressure than females (all *P* < 0.05). There were no significant differences in age, BMI, and HR between males and females (*P* > 0.05).

**Table 1:** Baseline characteristics of the population.

**Parameters**	**Total (*n* = 183)**	**Male (*n* = 96)**	**Female (*n* = 87)**	***P*-value**
Age (y)	10.0 (6.0, 13.0)	10.0 (6.0, 14.0)	8.0 (6.0, 13.0)	0.06
Height (cm)	142.0 (122.0, 160.0)	150 (124.5, 166.8)	135.0 (120.0, 152.0)	0.002
Weight (kg)	39.0 (25.0, 50.0)	41 (46.0, 57.1)	34.0(25.0, 46.0)	0.01
BSA (m^2^)	1.2 ± 0.4	1.3 ± 0.4	1.1 ± 0.4	0.01
BMI (kg/m^2^)	18.7 ± 3.4	19.1 ± 3.7	18.3 ± 3.0	0.10
HR (bpm)	85.9 ± 9.4	85.2 ± 9.4	86.7 ± 0.4	0.29
SBP (mm Hg)	100.9 ± 2.4	103.4 ± 13.2	98.1 ± 10.8	0.003
DBP (mm Hg)	64.3 ± 8.6	66.3 ± 8.7	62.0 ± 7.9	0.001

*Data are expressed as mean ± SD, or median (IQR). BMI, body mass index; BSA, Body surface area; HR, Heart rate; SBP, Systolic blood pressure; DBP, Diastolic blood pressure*.

### Myocardial Work Measurements

As shown in Table 2, for the total study population, the mean for GWI was 1,448.7 ± 265.0 mm Hg%, 1,859.8 ± 290.7 mm Hg% for GCW, and the median (interquartile range) for GWW was 54.0 (33.0–82.0) mm Hg% and 97.0 (95.0–99.0) % for GWE. Males had greater GWI and GCW than females (1,572.5 ± 250.2 mm Hg% vs. 1,312.2 ± 208.7 mm Hg% and 1,944.3 ± 299.2 mm Hg% vs. 1,766.6 ± 251.5 mm Hg%, respectively, all *P* < 0.001). There were no statistically significant differences in GWW, or GWE between males and females (*P* > 0.05).

**Table 2:** Myocardial work indices obtained from the population.

**Parameters**	**Total (*n* = 183)**	**Male (*n* = 96)**	**Female (*n* = 87)**	***P*-value**
GWI (mm Hg%) 95% confidence interval	1448.7 ± 265.0 929.3–1968.1	1572.5 ± 250.2 1082.1–2062.9	1312.2 ± 208.7 903.1–1721.3	<0.001
GCW (mm Hg%) 95% confidence interval	1859.8 ± 290.7 1290.0–2429.6	1944.3 ± 299.2 1357.9–2530.7	1766.6 ± 251.5 1273.7–2259.5	<0.001
GWW (mm Hg%) 5–95° percentile	54.0 (33.0, 82.0) 16.4–157.4	55.0 (35.3, 83.8) 18.3–179	51.0 (32.0, 79.0) 15.4–145	0.32
GWE (%) 5–95° percentile	97.0 (95.0–99.0) 92–99	97.0 (95.0-98.0) 91.9–98.2	96.0 (95.0–98.0) 91.4–99	0.89

*Data are expressed as mean ± SD, or median (IQR). GWI, golbal work index; GCW, global constructive work; GWW, global wasted work; GWE, global work efficiency*.

### Myocardial Work Indices in Different age Subgroups

**Figure 2:**
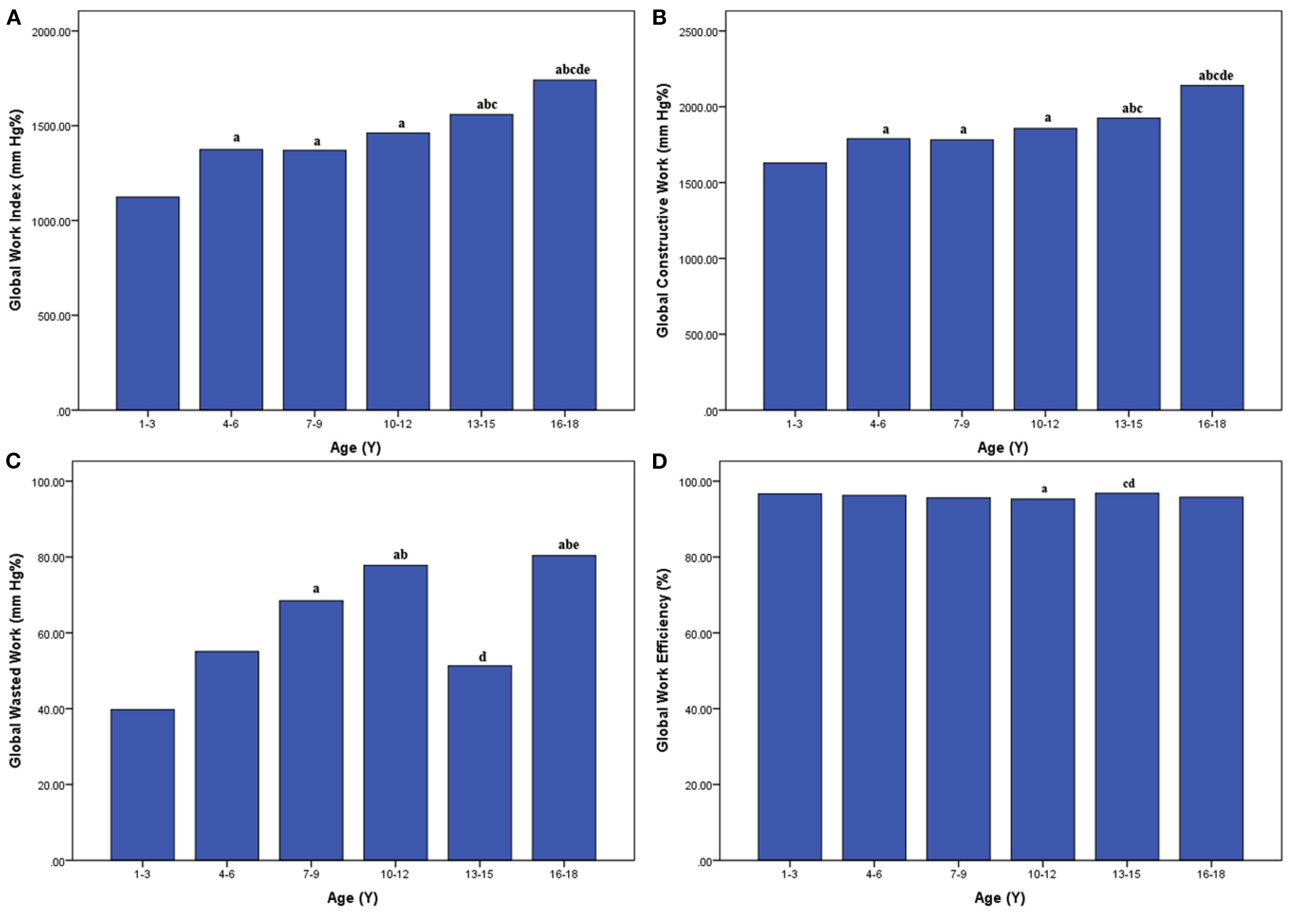
Bar graphs showing average global work index **(A)**, global constructive work **(B)**, global wasted work **(C)**, and global work efficiency **(D)** in different age subgroups. ^a^*P* < 0.05 vs. age 1–3 years; ^b^*P* < 0.05 vs. age 4–6 years; ^c^*P* < 0.05 vs. age 7–9 years; ^d^*P* < 0.05 vs. age 10–12 years; ^e^*P* < 0.05 vs. age 13–15 years.

As displayed in Figure 2 and Table 3, there were significant differences in myocardial work indices among six age subgroups (*P* < 0.05). GWI, GCW were the lowest in the subgroup 1–3 years and the highest in the subgroup 16–18 years, while there were no significant differences among subgroup 4–6, 7–9, and 10–12 years (*P* > 0.05). In the subgroup 10–12 years, GWW was higher and thus GWE was lower than that in the subgroup 1–3 years (*P* < 0.05). In the subgroup 13–15 years, GWW was lower and thus GWE was higher than that in the subgroup 10–12 years (*P* < 0.05).

**Table 3:** Values of myocardial work indices in different age subgroups.

Variables	1–3 y *n* = 23	4–6 y *n* = 31	7–9 y *n* = 34	10–12 y *n* = 34	13–15 y *n* = 32	16–18 y *n* = 29	*P*-value
GWI, mm g%	1123.9 ± 110.6	1374.6 ± 202.8^a^	1370.4 ± 187.6^a^	1461.9 ± 250.8^a^	1558.8 ± 204.6^abc^	1740.9 ± 195.4^abcde^	<0.001
GCW, mm Hg%	1629.3 ± 211.6	1789.4 ± 236.1^a^	1782.2 ± 223.9^a^	1856.7 ± 262.6^a^	1925.5 ± 248.9^abc^	2140.0 ± 320.9^abcde^	<0.001
GWW, mm Hg%	37 (22, 58)	54 (38, 72)	56.5 (28.3, 92.8)^a^	77 (38.8, 101.8)^ab^	48 (33.5, 66.8)^d^	59 (33, 104.5)^abe^	0.01
GWE, %	97(96, 98)	97 (95, 97)	96 (94, 98)	96 (94, 98)^a^	97 (96, 98)^cd^	97 (94, 98)	0.04

*Data are expressed as mean ± SD, or median (IQR)*.

a *P < 0.05 vs. age 1–3 years*;

b *P < 0.05 vs. age 4–6 years*;

c *P < 0.05 vs. age 7–9 years*;

d *P < 0.05 vs. age 10–12 years*;

e *P < 0.05 vs. age 13–15 years. Abbreviations as Table 2*.

### Relationship Between Myocardial Work Indices and Baseline Parameters

Correlations between myocardial work indices and baseline parameters are shown in Supplementary Table S1. GWI was strongly correlated with age, sex, height, weight, BMI, BSA, systolic blood pressure (SBP), and diastolic blood pressure (DBP) (correlation coefficient 0.63, 0.51, 0.61, 0.61, 0.51, 0.64, 0.62, and 0.61, respectively, all *P* < 0.001), and moderately correlated with HR (correlation coefficient −0.46, *P* < 0.001). GCW was strongly correlated with BSA (correlation coefficient 0.51, *P* < 0.001), and moderately correlated with age, sex, height, weight, BMI, HR, SBP, and DBP (correlation coefficient 0.48, 0.32, 0.48, 0.50, 0.43, −0.38, 0.47, and 0.44, respectively, all *P* < 0.001). GWW was weakly correlated with age and SBP (correlation coefficient 0.15, and 0.13, respectively, *P* < 0.05). There were no significant correlations between GWE and the baseline parameters (*P* > 0.05).

**Figure 3:**
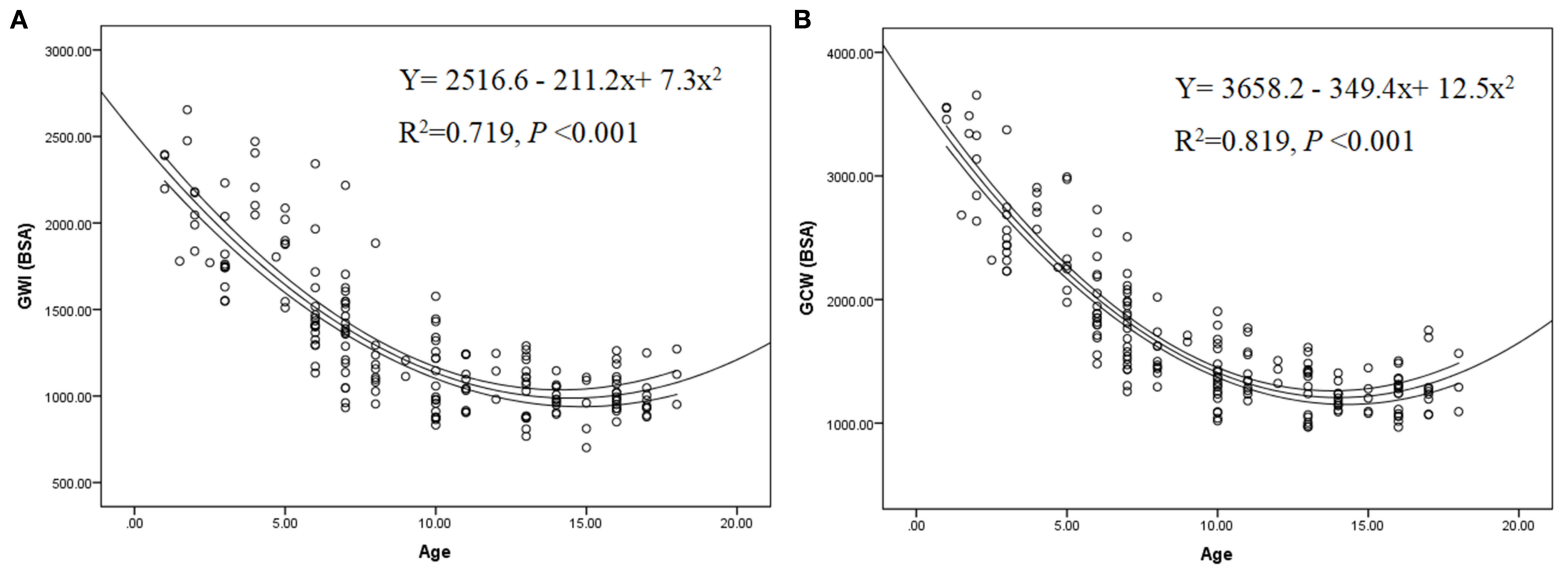
Impact of age on GWI (BSA) **(A)**, GCW (BSA) **(B)**, Lines represent mean and 95% confidence interval. GWI (BSA), GCW (BSA) indicate global work index, global constructive work indexed to body surface area.

Considering the interdependence between the baseline parameters and strong correlations of age and BSA with GWI, GCW, these myocardial work indices were indexed to BSA: GWI (BSA), GCW (BSA), and GWW (BSA). We found significant correlations between GWI (BSA), GCW (BSA) and age (correlation coefficient −0.77, and −0.81, respectively, *P* < 0.001) (Figure 3), with the following equations:


$$\begin{array}{c} GWI\left(BSA\right)=2516.6-211.2\times Age+7.3\times Age^{2}\\ GCW\left(BSA\right)=3658.2-349.4\times Age+12.5\times Age^{2} \end{array}$$

### Reproducibility

**Figure 4:**
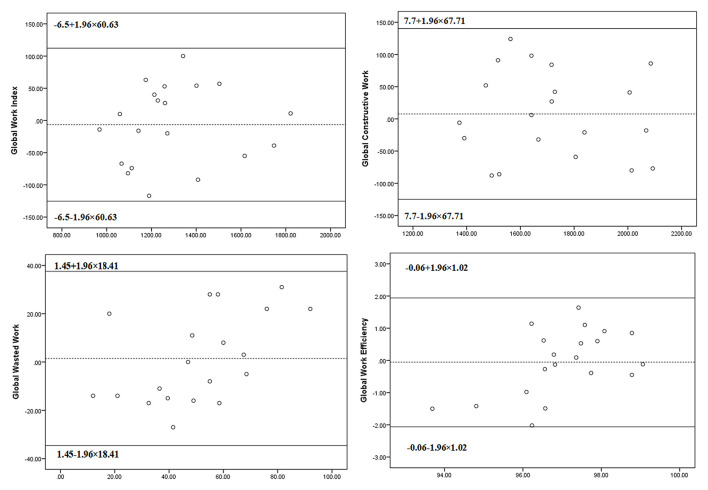
The Bland–Altman analysis for assessing intra-observer variability of global work index, global constructive work, global wasted work, and global work efficiency. Lines represent bias and 95% limits of agreement for measurements performed in 20 subjects.

**Figure 5:**
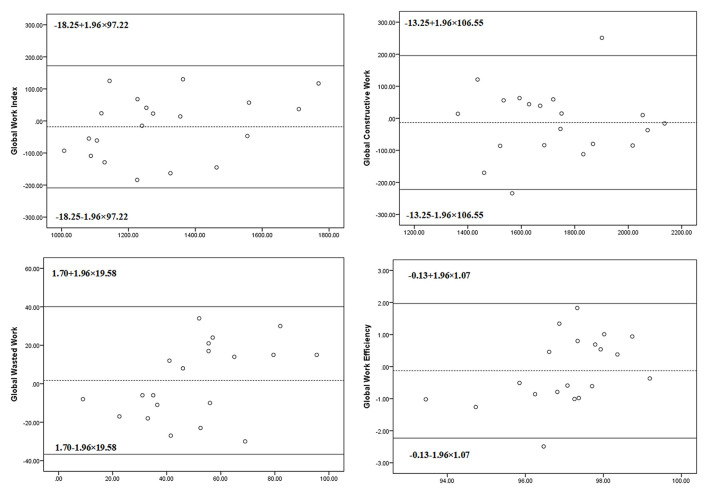
The Bland–Altman analysis for assessing inter-observer variability of global work index, global constructive work, global wasted work, and global work efficiency. Lines represent bias and 95% limits of agreement for measurements performed in 20 subjects.

Intra-observer and inter-observer analyses showed good reproducibility for measurements of myocardial indices. The ICCs for the intra-observer variability were 0.97 (95% CI: 0.92–0.99, *P* < 0.001) for GWI, 0.96 (95% CI: 0.90–0.98, *P* < 0.001) for GCW, 0.81 (95% CI: 0.53–0.93, *P* < 0.001) for GWW, and 0.87 (95% CI: 0.66–0.95, *P* < 0.001) for GWE. The ICCs for the inter-observer variability were 0.90 (95% CI: 0.78–0.96, *P* < 0.001) for GWI, 0.90 (95% CI: 0.76–0.96, *P* < 0.001) for GCW, 0.79 (95% CI: 0.46–0.92, *P* = 0.001) for GWW, and 0.79 (95% CI: 0.45–0.92, *P* = 0.001) for GWE. Bland-Altman plots for intra- and inter-observer variability are shown in Figures 4, 5. The mean intra-observer variability for GWI, GCW, GWW, and GWE was −6.5 mm Hg, 7.7 mm Hg, 1.45 mm Hg, and −0.06%, respectively. The mean inter-observer variability for GWI, GCW, GWW, and GWE was −18.25 mm Hg, −13.25 mm Hg, 1.70 mm Hg, and −0.13%, respectively).

## Discussion

In the present study, we provided the normal ranges of the myocardial work indices, including GWI, GCW, GWW, and GWE, in healthy children derived from PSL analysis. GWI and GCW were significantly correlated with baseline parameters, including age, height, weight, BSA, BMI, HR, SBP, and DBP. In this study, we also provide equations for the variation of GWI and GCW, indexed to BSA, with age. Additionally, our results showed a good reproducibility for the assessment of myocardial work indices in children.

### Normal Reference Values of Noninvasive Myocardial Work Indices

Previous studies (10–14) have proposed the reference values of noninvasive myocardial work indices in adults and children. The European Association of Cardiovascular Imaging (EACVI) Normal Reference Ranges for Echocardiography (NORRE) study provided for the first time the reference values of myocardial work indices of healthy individuals with a mean age of 45 ± 13 years. The reference values of GWI and GCW were 1,896 ± 308 mm Hg% (95% CI: 1,292–2,505) and 2,232 ± 331 mm Hg% (95% CI: 1,582–2,881), respectively. The reference ranges of GWW and GWE were 78.5 (53–122.2) mm Hg% and 96 (94–97)%, respectively (10). Morbach et al. (11) and Galli et al. (12) presented analyses on the reference values of healthy people from the Characteristics and Course of Heart Failure STAges A/B and Determinants of Progression (STAAB) cohort study and a study at the University Hospital of Rennes, respectively. The reference values of GWI, GCW and GWW proposed by Morbach et al. and Galli et al. were higher than those proposed by EACVI, while the reference value of GWE was similar.

Pham et al. (13) provided the reference values of noninvasive myocardial work indices in children aged 4–21 years. The mean GWI was 1688 ± 219 mm Hg% and mean GWE was 96.5 ± 10.4%. The mean GCW was 1959 ± 207 mm Hg% and the mean GWW of 61.1 ± 30.9 mm Hg%. Tretter et al. (14) proposed normal values of noninvasive myocardial work indices in healthy adolescents at rest. The reference values of GWI, GCW and GWW proposed by Tretter et al. were higher than those proposed by Pham et al., while the reference value of GWE was similar. In our study, we enrolled subjects aged 1–18 years, the reference values of GWI GCW, GWW and GWE were 1,448.7 ± 265.0 mm Hg%, 1,859.8 ± 290.7 mm Hg%, 54.0 (33.0–82.0) mm Hg% and 97 (95–98)%, respectively. The reference values of GWI, GCW and GWW of children were lower than those proposed by previous studies (10–14), while the reference value of GWE was similar to that of in earlier studies. These differences can be explained by factors as a single center study, sample size and race of subjects.

### Relationship Between Myocardial Work Indices and Baseline Parameters

As previously reported (19), myocardial work is derived from longitudinal strain and LV pressure. First, the segmental strain rate (expressed as %/s) was obtained by differentiating the segmental strain (%). The segmental strain rate was multiplied by the instantaneous LV pressure (mm Hg) to obtain the instantaneous power (mm Hg%/s). Finally, the instantaneous power was integrated over time to obtain myocardial work (mm Hg%). LV pressure was estimated from brachial artery cuff pressure. Therefore, myocardial work was closely related to longitudinal strain and brachial artery cuff pressure.

Previous studies (20, 21) have confirmed the effects of growth parameters, including age and BSA, on longitudinal strain in healthy children. Pham et al. (13) found that GWI and GCW were correlated with BSA and SBP, and no significant difference was found in myocardial work indices across age groups and gender in children, while Tretter et al. (14) demonstrated that GWI and GCW were associated with age, SBP, and DBP in healthy adolescents. Morbach et al. (11) found that higher levels of blood pressure correlated with higher GCW, GWI, and GWW, resulting in lower GWE in adults. In our study, we found that GWI and GCW were significantly correlated with age, sex, height, weight, BSA, BMI, blood pressure and HR in children. All these studies showed that myocardial work indices were related to blood pressure. The incongruent findings might be due to sample differences, including sample size, the distribution of age and sex. Perhaps these findings can be explained by higher blood pressure, BSA, or BMI of males than those of females and by the significant associations between height, weight, BSA, BMI, blood pressure, HR and age in the study.

Because these baseline parameters were interdependent, it is important to analyze which ones are the most important factors. For GWI and GCW, we found that BSA and age had a great impact. Interestingly, after indexed to BSA, we found that GWI (BSA) and GCW (BSA) remained significantly negatively correlated with age, indicating that the myocardial work of younger children was higher than that of older children after BSA correction. We also provided the equations of GWI (BSA) and GCW (BSA) with age, which provided references for the evaluation of myocardial work in healthy children.

### Limitations

Our study had the following limitations. This study was a single center study with a limited number of healthy subjects. The number of subjects, especially females, was insufficient for stratified analysis by age and sex, and sample size needs to be expanded in further studies. In this study, we excluded subjects younger than 12 months, because rapid growth and increased HR in infants may have a significant impact on LV myocardial tension and myocardial function. Furthermore, we used brachial artery cuff pressure for the measurement of myocardial work indices. In children, there may be some fluctuations in blood pressure. Repeating the measurement of blood pressure can improve the accuracy of measurements of myocardial work indices.

## Conclusions

Here, we proposed the normal reference values and regression equations for GWI and GCW based on age and BSA in healthy children. This might provide a basis of reference for the evaluation of cardiac function in children with cardiopulmonary disease. In further studies, we will recruit healthy adults and aim to propose reference values corrected for all ages in healthy people >1–year-old.

## Data Availability Statement

The original contributions presented in the study are included in the article/Supplementary Material, further inquiries can be directed to the corresponding author/s.

## Ethics Statement

The studies involving human participants were reviewed and approved by the Ethics Committee of Fuwai Central China Cardiovascular Hospital. Written informed consent to participate in this study was provided by the participants' legal guardian/next of kin. Written informed consent was obtained from the individual(s), and minor(s)' legal guardian/next of kin, for the publication of any potentially identifiable images or data included in this article.

## Author Contributions

CC, LL, and LZ designed the study, analyzed data, wrote, reviewed, and edited the manuscript. QZ, YL, DH, YH, and YW performed this study. All authors contributed to the article and approved the submitted version.

## Funding

This study was supported by National Natural Science Foundation of China (82071950), National Natural Science Foundation of Henan for Excellet Young Scientists (202300410364), Medical Science and Technology Project of Henan Province (SB201901099), and Henan Provincial Medical Science and Technology Research Project (LHGJ20190805, LHGJ20200084).

## Conflict of Interest

The authors declare that the research was conducted in the absence of any commercial or financial relationships that could be construed as a potential conflict of interest.

## Publisher's Note

All claims expressed in this article are solely those of the authors and do not necessarily represent those of their affiliated organizations, or those of the publisher, the editors and the reviewers. Any product that may be evaluated in this article, or claim that may be made by its manufacturer, is not guaranteed or endorsed by the publisher.
