# Supplementary material for: Reference Values of Noninvasive Myocardial Work Indices Measured by Echocardiography in Healthy Children
Source: Front Pediatr. 2022 Jun 16;10:792526. doi: 10.3389/fped.2022.792526 (PMC9243532; doi:10.3389/fped.2022.792526)
Supplement: Supplementary file 1 [file Table_1.DOC]

**Supplemental Table 1** Correlation analysis between myocardial work indices and baseline characteristics

| Variables | GWI | | GCW | | GWW | | GWE | |
| --- | --- | --- | --- | --- | --- | --- | --- | --- |
|  | Coefficient | *P* value | Coefficient | *P* value | Coefficient | *P* value | Coefficient | *P*  value |
| Age | 0.63 | <0.001 | 0.48 | <0.001 | 0.15 | 0.04 | -0.04 | 0.64 |
| Sex | 0.51 | <0.001 | 0.32 | <0.001 | 0.07 | 0.32 | 0.01 | 0.89 |
| Height | 0.61 | <0.001 | 0.48 | <0.001 | 0.14 | 0.06 | -0.05 | 0.55 |
| Weight | 0.61 | <0.001 | 0.50 | <0.001 | 0.13 | 0.09 | 0.01 | 0.87 |
| Body mass index | 0.51 | <0.001 | 0.43 | <0.001 | 0.11 | 0.16 | 0.03 | 0.62 |
| Body surface area | 0.64 | <0.001 | 0.51 | <0.001 | 0.13 | 0.08 | 0.01 | 0.87 |
| Heart rate | -0.46 | <0.001 | -0.38 | <0.001 | -0.08 | 0.30 | -0.01 | 0.88 |
| Systolic blood pressure | 0.62 | <0.001 | 0.47 | <0.001 | 0.15 | 0.04 | -0.02 | 0.79 |
| Diastolic blood pressure | 0.61 | <0.001 | 0.44 | <0.001 | 0.12 | 0.10 | -0.05 | 0.47 |

GWI, golbal work index; GCW, global constructive work; GWW, global wasted work; GWE, global work efficiency.
